# Sociodemographic, lifestyle, overall health and oral health factors associated with negative self-perception of oral health among the elderly: a cross-sectional study, Brazil, 2019

**DOI:** 10.1590/S2237-96222026v35e20250512.en

**Published:** 2025-11-28

**Authors:** Mariella Agostinho Gonçalves Lourenço, Flávio Pereira dos Santos, Bianca Souto de Medeiros Santos, Mateus Guedes Carvalho, Rafael Barroso Pazinatto, Fabíola Pessôa Pereira Leite, Laércio Almeida de Melo

**Affiliations:** 1 Universidade Federal de Juiz de Fora, Juiz de Fora, MG, Brazil; 2 Universidade Federal do Rio Grande do Norte, Departamento de Odontologia, Natal, RN, Brazil

**Keywords:** Oral Health, Elderly, Sociodemographic Factors, Life Style, Cross-Sectional Studies, Salud Bucal, Anciano, Factores Sociodemográficos, Estilo de Vida, Estudios Transversales

## Abstract

**Objective:**

To identify sociodemographic, lifestyle and overall health factors and variables related to oral health conditions associated with negative perception of oral health among older Brazilians.

**Methods:**

This was a cross-sectional, population-based study using data extracted from the 2019 Brazilian National Health Survey. Initially, the chi-square test was used for data analysis. Subsequently, multivariate Poisson regression analysis was performed to determine adjusted prevalence ratios.

**Results:**

The sample consisted of 22,728 older adults. Of the participants, 33.2% identified their oral health as poor or very poor. This negative self-perception, based on the multivariate analysis, was associated with male sex (p-value<0.001; prevalence ratio [PR] 1.03; 95% confidence interval [95%CI] 1.02; 1.04), Black people (p-value<0.001; PR 1.04; 95%CI 1.03; 1.05), illiterate people (p-value<0.001; PR 1.03; 95%CI 1.02; 1.05), those with multimorbidity (p-value<0.001; PR 1.03; 95%CI 1.02; 1.03), those who did not brush their teeth every day (p-value<0.001; PR 1.14; 95%CI 1.07; 1.22), those who did not have dental insurance (p-value<0.001; PR 1.05; 95%CI 1.04; 1.06), those who had difficulty eating (p-value<0.001; RP 1.34; 95%CI 1.31; 1.38) and those who were not completely edentulous (p-value<0.001; 1.04; 95%CI 1.03; 1.04).

**Conclusion:**

Negative self-perception of oral health in older adults is associated with poorer socioeconomic conditions, accumulation of chronic diseases in a single individual, poorer oral hygiene habits, difficulty eating, and presence of teeth in the mouth.

## Resumen

**Objetivo:**

Identificar factores sociodemográficas, de estilo de vida y de salud general y variables de salud bucal asociadas con percepciones negativas de salud bucal entre adultos mayores brasileños.

**Métodos:**

Estudio poblacional transversal con datos extraídos de la Encuesta Nacional de Salud de Brasil de 2019. Inicialmente, se utilizó la prueba de chi-cuadrado para el análisis de datos. Posteriormente, se realizó un análisis multivariado del tipo regresión múltiple de Poisson para determinar las razones de prevalencia ajustadas.

**Resultados:**

La muestra estuvo compuesta por 22.728 adultos mayores. De los participantes, el 33,2% identificó su salud bucal como mala o muy mala. Esta autopercepción negativa, basada en el análisis multivariado, se asoció con el sexo masculino (valor p<0,001; razón de prevalencia [RP] 1,03; intervalo de confianza del 95% [IC95%] 1,02; 1,04), personas de raza negra (valor p<0,001; RP 1,04; IC95% 1,03; 1,05), personas analfabetas (valor p<0,001; RP 1,03; IC95% 1,02; 1,05), personas con multimorbilidad (valor p<0,001; RP 1,03; IC95% 1,02; 1,03), personas que no se cepillan los dientes todos los días (valor p<0,001; RP 1,14; IC95% 1,07; 1,22), personas que no tienen seguro dental (valor p<0,001; RP 1,05; IC95% 1,04; 1,06), los que tienen dificultad para alimentarse (valor p<0,001; RP 1,34; IC95% 1,31; 1,38) y los que no están completamente edéntulos (valor p<0,001; 1,04; IC95% 1,03; 1,04).

**Conclusión:**

La autopercepción negativa de la salud bucal en adultos mayores se asocia con peores condiciones socioeconómicas, acumulación de enfermedades crónicas en un mismo individuo, peores hábitos de higiene bucal, dificultad para comer y la presencia de dientes en la boca.

**Ethical aspects**

This research used public domain anonymized databases.

## Introduction 

The definition of what is considered elderly varies globally according to chronological age. Generally, being 60 and over, or 65 and over, is used as a reference to classify this population group (1). Regardless of these differences in classification, there is rapid growth in the elderly population worldwide, especially among those aged 80 and over (2). This rapid increase has negative repercussions for both overall health and oral health, resulting in high costs for complex and difficult-to-access procedures. Therefore, it is essential to understand which health standards can be considered adequate, including oral health, as a strategy for promoting well-being and improving quality of life among the elderly (3).

Among the criteria considered for promoting active aging, oral health is a relevant element (4). However, it can be seen that a significant part of the elderly population faces difficulties in achieving healthy aging. As age advances, both oral tissues and teeth present unfavorable structural and functional changes (5-7). The concept of oral health, according to the World Dental Federation, encompasses aspects such as chewing, swallowing, and the presence of pain and discomfort (8). These factors, when associated with painful experiences related to teeth and gums, as well as limitations during chewing, can significantly compromise the quality of life of the elderly (8-10).

In the context of oral health, self-perception is defined as an individual’s subjective ability to recognize and assess their own oral health status, considering their personal experiences, impact on daily functions and repercussions on social interaction (4,11). Existing literature on oral health self-perception among elderly populations, for the most part, establishes a correlation between self-perception and clinical or sociodemographic variables, partially disregarding the influence of potentially more determinant factors, such as lifestyle, overall health status and oral hygiene habits (12-14). Evidence also indicates that many elderly individuals who report positive self-perception of oral health have unfavorable clinical conditions. Therefore, it can be inferred that extraclinical factors may play a more relevant role in shaping this subjective perception (12-14).

Despite the extensive scientific literature on self-perceived oral health among the elderly, many studies are limited to analyzing clinical and sociodemographic aspects, disregarding subjective factors that can significantly influence how the elderly perceive their health (12-14). Self-perceived health, in general, is a complex phenomenon, influenced by multiple dimensions, such as functional conditions, presence of pain, previous experiences, limitations in daily life, and even cultural factors (15,16). Theoretical models developed in the field of public health indicate that the way in which health is self-rated results from interaction between objective and subjective conditions and can reflect broader aspects of an individual’s life, such as functionality and social integration (15-17). In the field of oral health, there are still few studies that address this complexity and take into consideration, in an integrated manner, specific oral factors (such as tooth loss, pain, chewing difficulties), lifestyle, systemic morbidities and social determinants in the building of subjective health perceptions. Therefore, identifying factors associated with negative self-rated oral health among the elderly, considering multiple dimensions, can represent a relevant strategy for promoting healthy behaviors and supporting the planning of public health initiatives focused on aging. As such, this study aims to investigate association of sociodemographic factors, lifestyle habits, overall health conditions and oral aspects with negative oral health perceptions among elderly Brazilians. Given their population coverage, the results obtained can offer relevant subsidies for the formulation of public policies aimed at the oral health of elderly people in Brazil, as well as in other countries with similar socioeconomic contexts.

## Methods 

### Design 

This study has a cross-sectional, population-based design. We used data from the most recent edition of the National Health Survey, conducted in Brazil in 2019.

### Setting 

The 2019 National Health Survey, conducted by the Brazilian Institute of Geography and Statistics in partnership with the Brazilian Ministry of Health, aimed to provide updated information on the health status of the Brazilian population. The survey included individuals aged 15 and over and investigated multiple aspects, including health status, lifestyles, access to and use of health services, disease prevalence, medication use and mental health. Furthermore, it provided an overview of the population’s living conditions, highlighting regional and socioeconomic inequalities. Data collection took place between August 2019 and March 2020.

### Participants 

The National Health Survey was conducted by means of interviews with people aged 15 years or older living in households located in both urban and rural areas, covering all five macro-regions of the country. Our study, however, deals solely with the elderly population, defined as individuals aged 60 years or older, who answered the question regarding self-perceived oral health. Details regarding the sample size calculation are described in the National Health Survey methodology (18).

### Variables 

Identification of elderly individuals who negatively self-rated their oral health, was based on those who subjectively reported that they perceived their oral health as “poor” or “very poor” by answering the question: “In general, how do you rate your oral health (teeth and gums)?” Elderly individuals who opted to answer “very good,” “good,” or “fair” were categorized as having a positive self-perception of oral health.

The sociodemographic variables used were sex, age, race/skin color, marital status, education level, health insurance and dental insurance. Lifestyle variables analyzed included tobacco smoking and alcohol consumption. The variable analyzed as an indicator of the overall health of the elderly was the presence of two or more chronic diseases in the same individual (multimorbidity). Variables related to oral health included toothbrushing frequency, edentulism and eating difficulties due to oral problems. All independent variables were taken from the National Health Survey questionnaire.

### Data sources and measurement

This study used secondary information from the National Health Survey, conducted in Brazil in 2019. This information is available at: https://www.pns.icict.fiocruz.br/bases-de-dados/ (19). In addition, the database used in this research is available at: https://drive.google.com/file/d/1VDjasU9Wrteyqfme5LmeCIAX45Z0QrXs/view?usp=sharing.

### Bias control

Bias control in this study was based on the National Health Survey sampling strategy, which considered the different selection probabilities and the specificities of the complex sampling design. To this end, sampling weights were assigned to the households and individuals included, with the final weight calculated from the inverse of the selection probabilities at each stage of the sampling plan, with additional adjustments for nonresponse and calibration based on population totals. Furthermore, because this was a cluster sample, appropriate statistical software was used, which incorporated the effects of stratification and data aggregation in the estimation of indicators and the determination of associated accuracy measures.

### Study size

The National Health Survey was conducted throughout Brazil, covering all its macro-regions, including urban and rural areas, as well as households in state capital cities and metropolitan regions. Through its broad coverage and detailed methodology, the survey offers a comprehensive overview of the health status of the Brazilian population.

### Statistical methods

Data analysis was performed using the Statistical Package for the Social Sciences (SPSS) software, version 22.0. Initially, the frequency distribution of the dependent and independent variables was calculated in order to create tables. The chi-square test was then used to analyze the relationship between negative self-perceived oral health and sociodemographic variables related to lifestyle, overall health status and oral health aspects.

Unadjusted prevalence ratios and their respective 95% confidence intervals were also calculated. Variables with a p-value<0.200 were selected for multicollinearity assessment. Considering the sample size, the multicollinearity criterion was defined as a p-value≤0.001 between independent variables, and those strongly associated with each other were not included simultaneously in the multivariate model. Multivariate analysis was conducted using Poisson multiple regression, with estimates of adjusted prevalence ratios and their respective 95% confidence intervals. Although the model yields the Incidence Rate Ratio, in cross-sectional studies with a prevalent outcome, this estimate has an interpretation equivalent to the prevalence ratio. All analyses considered the effect of the sampling design, post-stratification weights and nonresponse rates. 

## Results 

The study sample consisted of 22,728 elderly individuals, aged between 60 and 112 years, with a mean age of 70.1 years (±7.0). The majority of the participants were found to be female (56.7%), between 60 and 69 years old (55.4%), self-identified as White (51.3%), married (51.1%), literate (80.7%), did not have health insurance (70.7%), were non-smokers (88.1%), and did not consume alcoholic beverages (72.6%). In addition, 60.4% had two or more chronic diseases (multimorbidity), 99.6% reported daily tooth brushing, 91.9% did not have dental insurance, 96.5% did not report eating difficulties due to oral problems, and 68.0% were not fully edentulous.

Prevalence of negative self-perceived oral health was 33.2%. The distribution of the independent variables and their associations with self-perceived oral health in the univariate analysis are presented in Table 1. Considering this preliminary analysis and multicollinearity being found between the, “age”, “marital status”, “health insurance” and “use of alcoholic beverages” variables, they were excluded from the Poisson multiple regression model due to their high correlation with other factors. In the multivariate analysis, significant association was found between negative self-perceived oral health and males, self-identified Black individuals, illiterate elderly individuals, those with multimorbidity, those who did not brush their teeth daily, individuals without dental insurance, elderly individuals with eating difficulties, and those who were not fully edentulous (Table 2).

**Table 2:** Adjusted prevalence ratios (PR) and 95% confidence intervals (95%CI) of negative self-perception of oral health among elderly people, according to study variables (multivariate analysis). Brazil, 2019 (n=22,728)

Variable	PR (95%CI)	p-value
Sex	1.03	<0.001
Male	(1.02; 1.04)	
Female	1.00	
**Race/skin color^a^**		<0.001
White	1.00	
Brown	1.03 (1.02; 1.03)	
Black	1.04 (1.03; 1.05)	
Other	1.01 (1.00; 1.02)	
Schooling	1.03	<0.001
Illiterate	(1.02; 1.05)	
Literate	1.00	
**Tobacco smoker**	1.00	0.736
Yes	(0.99; 1.02)	
No	1.00	
**Presence of multimorbidity^a^**	1.03	<0.001
Yes	(1.02; 1.03)	
No	1.00	
**Tooth brushing frequency^a^**	1.14	<0.001
Does not brush every day	(1.07; 1.22)	
At least once a day	1.00	
**Dental insurance**	1.05	<0.001
No	(1.04; 1.06)	
Yes	1.00	
**Difficulty in eating^a^**	1.34	<0.001
Severe or very severe	(1.31; 1.38)	
Little or none	1.00	
**Total tooth loss**	1.04	<0.001
No	(1.03; 1.05)	
Yes		

^a^The difference in relation to the total number of cases (100.0%) corresponds to the number of cases recorded as unknown, left blank or missing on the National Health Survey database.

**Table 1:** Unadjusted prevalence ratios (PR) and 95% confidence intervals (95%CI) of negative self-perception of oral health among elderly people, according to study variables (bivariate analysis). Brazil, 2019 (n=22,728)

	Negative self-perception of oral health	Positive self-perception of oral health		
Variable	n (%)	n (%)	PR (95%CI)	p-value
Sex			1.10	<0.001
Male	3,932 (38.6)	7,509 (61.4)	(1.07; 1.13)	
Female	4,073 (32.5)	8,462 (67.5)	1.00	
**Age group** (years)				0.005
60-69	4,412 (35.1)	8,143 (64.9)	1.00	
70-79	2,469 (34.5)	4,688 (65.5)	0.96 (0.93; 0.99)	
≥80	1,124 (37.3)	1,892 (62.7)	1.03 (0.99; 1.07)	
**Race/skin color^a^**			1.00	<0.001
White	3,080 (31.1)	6,821 (68.9)		
Brown	3,785 (37.8)	6,216 (62.2)	1.11 (1.08; 1.14)	
Black	997 (40.6)	1,458 (59.4)	1.16 (1.10; 1.22)	
Other	142 (38.5)	227 (61.5)	1.10 (0.98; 1.24)	
**Marital status**			1.00	<0.001
Single	1,646 (39.1)	2,564 (60.9)		
Married	3,432 (34.5)	6,514 (65.5)	0.91 (0.87; 0.95)	
Divorced	859 (34.5)	1,629 (65.5)	0.91 (0.86; 0.96)	
Widowed	2,068 (34.0)	4,016 (66.0)	0.90 (0.86; 0.94)	
Schooling			1.29	<0.001
Illiterate	2,314 (43.0)	3,069 (57.0)	(1.21; 1.37)	
Literate	5,691 (32.8)	11,654 (67.2)	1.00	
**Health insurance**			1.46	<0.001
No	6,526 (38.6)	10,365 (61.4)	(1.35; 1.57)	
Yes	1,479 (25.3)	4,358 (74.7)	1.00	
**Tobacco smoker**			1.09	0.050
Yes	1,025 (38.2)	1,655 (61.8)	(1.00; 1.91)	
No	6,980 (34.8)	13,068 (65.2)	1.00	
**Use of alcoholic beverage**			1.05	0.017
Yes	1,835 (32.6)	3,797 (67.4)	(1.01; 1.09)	
No	6,170 (36.1)	10,926 (63.9)	1.00	
**Presence of multimorbidity^a^**			1.20	<0.001
Yes	4,665 (37.0)	7,953 (63.0)	(1.13; 1.27)	
No	2,913 (32.0)	6,194 (68.0)	1.00	
**Tooth brushing frequency^a^**			1.89	<0.001
Does not brush every day	78 (61.4)	49 (38.6)	(1.54; 2.31)	
At least once a day	7,343 (34.1)	14,171 (65.9)	1.00	
**Dental insurance**			1.34	<0.001
No	7,534 (36.0)	13,394 (64.0)	(1.19; 1.52)	
Yes	471 (26.2)	1,329 (73.8)	1.00	
**Difficulty in eating^a^**			2.63	<0.001
Severe or very severe	660 (75.8)	211 (24.2)	(2.45; 2.82)	
Little or none	6,154 (30.5)	14,023 (69.5)	1.00	
**Total tooth loss**			1.09	<0.001
No	5,654 (36.4)	9,879 (63.6)	(1.06; 1.12)	
Yes	2,351 (32.7)	4,844 (67.3)	1.00	

^a^The difference in relation to the total number of cases (100.0%) corresponds to the number of cases recorded as unknown, left blank or missing on the National Health Survey database.

## Discussion 

Perception of oral health as “poor” or “very poor” was associated with several individual characteristics among elderly Brazilians. This negative self-perception was more frequent among men, Black people, illiterate individuals, those with two or more chronic diseases, those without dental insurance, those who did not brush their teeth daily, those who still had teeth in their mouth, and those who reported difficulty eating. These findings highlight the influence of sociodemographic factors, systemic conditions, oral hygiene behaviors and functional limitations in shaping the subjective perception of oral health in this population.

Among the main strengths of this study is the use of population-based, nationally representative data, which confers external validity to the results. The adoption of robust analytical methods and adjustment for the complex sampling design increase the reliability of the findings. A limitation is the study’s cross-sectional design, which does not allow for the establishment of causal relationships. Even so, the data provide relevant insights that can support the implementation of preventive strategies aimed at reducing adverse oral health conditions among older adults in Brazil, as well as in other countries with similar socioeconomic and cultural profiles.

The prevalence of older adults who negatively assessed their oral health was 33.2%. This result is cause for concern, as oral changes at this stage of life not only compromise essential functions, such as chewing and nutrition, but also affect emotional, social and aesthetic aspects, significantly impacting quality of life (20-24). Compared to data from the 2010 SBBrasil oral health survey, in which 28.2% of older adults presented negative self-perception, this indicator has increased. This scenario reinforces the importance of intensifying preventive care programs and expanding the provision of specialized services aimed at caring for older adults.

The adjusted analysis showed that men tend to report a more negative perception of oral health than women. This may be associated with the fact that women, in general, adopt more health-conscious behaviors, attend medical and dental appointments more frequently, and value self-care practices. Men’s lower adherence to these services may explain their greater vulnerability to diseases such as caries and periodontitis (25). Furthermore, negative perceptions were more frequent among older Brown (Brazilian mixed race) and, especially, Black elderly people. This finding is supported by international studies that indicate strong association between racial inequalities and poorer oral health conditions (26). In particular, Black older adults are more likely to experience tooth loss compared to White older adults (26), a situation that may be exacerbated by less favorable socioeconomic conditions and reduced access to dental care.

Association between illiteracy and poor oral health perceptions has also been found. Lack of reading and writing skills can restrict understanding of preventive information and hinder adoption of self-care practices, increasing the risk of developing caries and periodontal disease (27-30). Cross-sectional studies in Brazil have shown that people with less education use dental services less frequently (29,31). Furthermore, older adults with low education levels are more likely to experience biofilm accumulation and oral mucosal changes, reflecting poor oral hygiene practices (25). Another aspect worth highlighting is low oral health literacy, which translates into difficulty understanding and applying oral care guidelines, which has been associated with oral health dissatisfaction (24). These factors indicate the need to develop educational strategies adapted to the cognitive and communicative abilities of the elderly population. 

Finally, we found that coexistence of two or more chronic diseases was related to a more negative perception of oral health. Multimorbidity is often associated with greater frailty, functional limitations and increased use of health services (32). In this context, the burden generated by multiple clinical conditions, combined with physical and psychological restrictions, can contribute to amplifying the negative perception of health in general, extending this judgment to oral health as well (30,32). 

Variables directly related to oral health were also associated with negative self-perception. Lack of dental insurance, lack of daily toothbrushing and having missing teeth stood out. In addition to reflecting unfavorable socioeconomic conditions, lack of dental insurance coverage limits access to health services, which can result in worsening oral health conditions and, consequently, a negative self-perception of oral health. The habit of not brushing daily demonstrates inadequate oral hygiene practices, increasing the likelihood of developing biofilm-dependent diseases such as caries and periodontitis, which directly impacts reported negative perception. Furthermore, presence of remaining teeth was also associated with an unfavorable assessment of oral health, possibly due to the occurrence of pain, since caries and periodontal disease are common among elderly individuals with natural teeth (10).

Eating difficulties resulting from oral health problems were strongly associated with negative self-perceptions of oral health. This limitation can result from tooth, muscle or joint pain, as well as poor teeth or poorly fitted dentures, compromising chewing function and eating comfort.

This set of findings highlights the interrelationship between social, behavioral, systemic and oral factors in the building of subjective perceptions of oral health among older adults. National and international studies demonstrate that this perception is sensitive to social inequalities, barriers to access to health services and oral health literacy levels (24,30). In different cultural contexts, as observed in studies conducted in Brazil and other Latin American countries, socioeconomic and functional factors are consistent determinants of negative self-perception (30,33).

The results of this study reinforce the need to strengthen dental care within the Brazilian National Health System, with emphasis on expanding access to dentures, educational initiatives and home care. Intersectoral strategies that integrate oral health care with healthy aging can help reduce inequalities and improve subjective and objective oral health indicators among the Brazilian elderly population (23,33).

In conclusion, the findings of this study suggest that negative self-perception of oral health among older adults in Brazil may be associated with unfavorable socioeconomic conditions and the presence of multiple chronic diseases. Furthermore, factors related to oral health may also contribute to this negative perception, such as inadequate hygiene practices, difficulty eating due to unhealthy oral conditions and the presence of remaining teeth, these being factors that may possibly have been associated with previous experiences of pain.

## References

[1] United Nations, Department of Economic and Social Affairs, Population Division (2019). World population ageing 2019.

[2] United Nations (2017). Monitoring of population programmes, focusing on changing population age structures and sustainable development, in the context of the full implementation of the Programme of action of the International Conference on Population and Development: report of the Secretary-General [Internet].

[3] Kreve S, Ávila GC, Santos LO, Reis AC (2020). Self-perception of oral health in older adults. Clin Lab Res Den.

[4] Henricsson S, Bengtsson VW, Renvert S, Berglund JS, Lundegren N, Andersson P (2024). Self-perceived oral health and orofacial appearance in an adult population, 60 years of age. Int J Dent Hyg.

[5] Schimmel M, Katsoulis J, Genton L, ller F (2015). Masticatory function and nutrition in old age. Swiss Dent J.

[6] Lamster IB, Asadourian L, Del Carmen T, Friedman PK (2000). The aging mouth: differentiating normal aging from disease. Periodontol 2000.

[7] Emami E, Souza RF, Kabawat M, Feine JS (2013). The impact of edentulism on oral and general health. Int J Dent.

[8] Glick M, Williams DM, Kleinman DV, Vujicic M, Watt RG, Weyant RJ (2016). A new definition for oral health developed by the FDI World Dental Federation opens the door to a universal definition of oral health. Int Dent J.

[9] Baiju RM, Peter E, Varghese NO, Sivaram R (2017). Oral health and quality of life: current concepts. J Clin Diagn Res.

[10] Müller F, Shimazaki Y, Kahabuka F, Schimmel M (2017). Oral health for an ageing population: the importance of a natural dentition in older adults. Int Dent J.

[11] Locker D, Clarke M, Payne B (2000). Self-perceived oral health status, psychological well-being, and life satisfaction in an older adult population. J Dent Res.

[12] Melo LA, Sousa MM, Medeiros AKB, Carreiro AFP, Lima KC (2016). Factors associated with negative self-perception of oral health among institutionalized elderly. Cien Saude Colet.

[13] Farias IPS, Montenegro LAS, Araújo EGO, Raymundo MLB, Brito ACM, Lucena EHG (2021). Impact of oral health on nutritional status, self-perception of oral health and quality of life of institutionalized elderly. J Clin Exp Dent.

[14] Esmeriz CEC, Meneghim MC, Ambrosano GMB (2012). Self-perception of oral health in non-institutionalised elderly of Piracicaba city, Brazil. Gerodontology.

[15] Lima-Costa MF, Firmo JOA, Uchôa E (2004). A estrutura da auto-avaliação da saúde entre idosos: projeto Bambuí. Rev Saude Pública.

[16] Pagotto V, Bachion MM, Silveira EA (2013). Autoavaliação da saúde por idosos brasileiros: revisão sistemática da literatura. Rev Panam Salud Publica.

[17] Pavão ALB, Werneck GL, Campos MR (2013). Autoavaliação do estado de saúde e a associação com fatores sociodemográficos, hábitos de vida e morbidade na população: um inquérito nacional. Cad Saude Publica.

[18] Stopa SR, Szwarcwald CL, Oliveira MM, Gouvea ECDP, Vieira MLFP, Freitas MPS (2020). National Health Survey 2019: history, methods and perspectives. Epidemiol Serv Saude.

[19] Fundação Oswaldo Cruz (2019). Pesquisa Nacional de Saúde (PNS) 2019.

[20] Algra Y, Haverkort E, Kok W, van Etten-jamaludin F, van Schoot L, Hollaar V (2021). The Association between Malnutrition and Oral Health in Older People: A Systematic Review. Nutrients.

[21] Hussein S, Kantawalla RF, Dickie S, Suarez-Durall P, Enciso R, Mulligan R (2022). Association of Oral Health and Mini Nutritional Assessment in Older Adults: A Systematic Review with Meta-analyses. J Prosthodont Res.

[22] Kato T, Umezaki Y, Naito T (2018). Effects of dropping out of dental treatment on the oral health-related quality of life among middle-aged subjects using web research. PLoS One.

[23] Silva MA, Batista AUD, Abreu MHNG, Forte FDS (2019). Oral Health Impact Profile: need and use of dental prostheses among Northeast Brazilian independent-living elderly. Cien Saude Colet.

[24] Tenani CF, Checchi MHR, Bado FMR, Ju X, Jamieson L, Mialhe FL (2020). Influence of oral health literacy on dissatisfaction with oral health among older people. Gerodontology.

[25] Oliveira MB, Lopes FF, Rodrigues VP, Alves CMC, Hugo FN (2018). Association between socioeconomic factors, behavioral, general health and oral mucosa status in elderly. Cien Saude Colet.

[26] Bastos JL, Constante HM, Schuch HS, Haag DG, Jamieson LM (2022). How do state-level racism, sexism, and income inequality shape edentulism-related racial inequities in contemporary United States? A structural intersectionality approach to population oral health. J Public Health Dent.

[27] Agostinho ACMG, Campos ML, Silveira JLGCD (2015). Edentulismo, uso de prótese e autopercepção de saúde bucal entre idosos. Rev Odontol UNESP.

[28] Farmer J, Phillips RC, Singhal S, Quiñonez C (2017). Inequalities in oral health: Understanding the contributions of education and income. Can J Public Health.

[29] Moreira RS, Nico LS, Tomita NE, Ruiz T (2005). A saúde bucal do idoso brasileiro: revisão sistemática sobre o quadro epidemiológico e acesso aos serviços de saúde bucal. Cad Saude Publica.

[30] Zanesco C, Bordin D, Santos CB, Müller EV, Fadel CB (2018). Factors determining the negative perception of the health of Brazilian elderly people. Rev Bras Geriatr Gerontol.

[31] Mendes DC, Poswar FO, Oliveira MVM, Haikal DS, Silveira MF, Martins AMEBL (2012). Analysis of socio-demographic and systemic health factors and the normative conditions of oral health care in a population of the Brazilian elderly. Gerodontology.

[32] Melo LA, Lima KC (2020). Prevalence and factors associated with multimorbidities in Brazilian older adults. Cien Saude Colet.

[33] Santos ASF, Lima RFR, Ferreira RC, Alencar GP, Carreiro DL, Silveira MF (2022). Use of oral health services among elderly Brazilians: mediation by tooth loss. Cien Saude Colet.

