# Supplementary material for: Sociodemographic, lifestyle, overall health and oral health factors associated with negative self-perception of oral health among the elderly: a cross-sectional study, Brazil, 2019
Source: Epidemiol Serv Saude. 2025 Nov 28;35:e20250512. doi: 10.1590/S2237-96222026v35e20250512.en (PMC12662408; doi:10.1590/S2237-96222026v35e20250512.en)
Supplement: Translation

# Translation: Fatores sociodemográficos, de estilo de vida, de saúde geral e bucal associados à autopercepção negativa de saúde bucal em idosos: estudo transversal, Brasil, 2019

**DOI:** 10.1590/S2237-96222026v35e20250512.pt

**Authors:** Mariella Agostinho Gonçalves Lourenço, Flávio Pereira dos Santos, Bianca Souto de Medeiros Santos, Mateus Guedes Carvalho, Rafael Barroso Pazinatto, Fabíola Pessôa Pereira Leite, Laércio Almeida de Melo

**Affiliations:** 1 Universidade Federal de Juiz de Fora, Juiz de Fora, MG, Brasil; 2 Universidade Federal do Rio Grande do Norte, Departamento de Odontologia, Natal, RN, Brasil

**Keywords:** Saúde Bucal, Idoso, Fatores Sociodemográficos, Estilo de Vida, Estudos Transversais

## Abstract

**Objetivo:**

Identificar fatores sociodemográficos, de estilo de vida, saúde geral e variáveis relacionadas às condições bucais que estão associados a uma percepção negativa de saúde bucal em idosos brasileiros.

**Métodos:**

Estudo transversal e de base populacional, com dados extraídos da Pesquisa Nacional de Saúde no Brasil de 2019. Inicialmente, o teste qui-quadrado foi utilizado para a análise dos dados. Em seguida, foi realizada uma análise multivariada do tipo regressão múltipla de Poisson para verificar as razões de prevalências ajustadas.

**Resultados:**

A amostra foi composta por 22.728 idosos. Dos participantes, 33,2% identificaram sua saúde bucal como ruim ou muito ruim. Essa autopercepção negativa, a partir da análise multivariada, esteve associada ao sexo masculino (p-valor<0,001; razão de prevalência [RP] 1,03; intervalo de confiança de 95% [IC95%] 1,02; 1,04), aos pretos (p-valor<0,001; RP 1,04; IC95% 1,03; 1,05), aos não alfabetizados (p-valor<0,001; RP 1,03; IC95% 1,02; 1,05), aos que possuem multimorbidade (p-valor<0,001; RP 1,03; IC95% 1,02; 1,03), aos que não escovam os dentes todos os dias (p-valor<0,001; RP 1,14; IC95% 1,07; 1,22), aos que não possuem plano odontológico (p-valor<0,001; RP 1,05; IC95% 1,04; 1,06), aos que têm dificuldade de se alimentar (p-valor<0,001; RP 1,34; IC95% 1,31; 1,38) e aos que não são edêntulos totais (p-valor<0,001; 1,04; IC95% 1,03; 1,04).

**Conclusão:**

A autopercepção negativa de saúde bucal em idosos está associada a piores condições socioeconômicas, acúmulo de doenças crônicas em um mesmo indivíduo, piores hábitos de higiene bucal, dificuldade de se alimentar e quando há ainda a presença de dentes em boca.

**Aspectos éticos**

Esta pesquisa utilizou bancos de dados de domínio público e anonimizados.

## Introdução 

A determinação do que se considera idoso varia globalmente conforme a idade cronológica. De forma geral, utiliza-se como referência a faixa etária de 60 ou 65 anos para classificar esse grupo populacional (1). Mesmo diante dessas diferenças, observa-se um crescimento acelerado da população idosa em todo o mundo, especialmente entre aqueles com 80 anos ou mais (2). Esse rápido aumento traz consigo repercussões negativas tanto para a saúde geral quanto para a saúde bucal, resultando em custos elevados com procedimentos complexos e de difícil acesso. Diante disso, torna-se essencial compreender quais padrões de saúde podem ser considerados adequados, incluindo a saúde oral, como estratégia para favorecer a promoção do bem-estar e o aprimoramento da qualidade de vida em pessoas idosas (3).

Entre os critérios considerados para a promoção de um envelhecimento ativo, a saúde bucal constitui um elemento relevante (4). Contudo, observa-se que uma parcela significativa da população idosa enfrenta dificuldades para alcançar um envelhecimento saudável. Com o avançar da idade, tanto os tecidos bucais quanto os dentes apresentam alterações estruturais e funcionais desfavoráveis (5-7). A concepção de saúde bucal, segundo a World Dental Federation, abrange aspectos como mastigação, deglutição, presença de dor e desconforto (8). Esses fatores, quando associados a experiências dolorosas relacionadas a dentes e gengivas, bem como a limitações durante a mastigação, podem comprometer significativamente a qualidade de vida dos idosos (8-10). 

No âmbito da saúde bucal, a autopercepção é definida como a capacidade subjetiva do indivíduo de reconhecer e avaliar sua própria condição bucal, considerando suas experiências pessoais, o impacto sobre funções cotidianas e as repercussões no convívio social (4,11). A literatura existente sobre o tema em populações idosas, em sua maioria, estabelece correlação entre a autopercepção e variáveis clínicas ou sociodemográficas, desconsiderando, em parte, a influência de aspectos potencialmente mais determinantes, como estilo de vida, condição de saúde geral e hábitos de higiene oral (12-14). Evidências apontam, inclusive, que muitos idosos que relatam uma autopercepção positiva de saúde bucal apresentam condições clínicas desfavoráveis. Dessa forma, infere-se que fatores extraclínicos podem exercer papel mais relevante na formação dessa percepção subjetiva (12-14). 

 Apesar da ampla produção científica sobre a autopercepção da saúde bucal em idosos, muitos estudos limitam-se à análise de aspectos clínicos e sociodemográficos, desconsiderando fatores subjetivos que podem exercer grande influência na forma como o idoso percebe sua saúde (12-14). A autopercepção da saúde, de maneira geral, é um fenômeno complexo, influenciado por múltiplas dimensões, como condições funcionais, presença de dor, experiências prévias, limitações no cotidiano e até fatores culturais (15,16). Modelos teóricos desenvolvidos no campo da saúde coletiva indicam que a autoavaliação da saúde resulta da interação entre condições objetivas e subjetivas e que pode refletir aspectos mais amplos da vida do indivíduo, como a funcionalidade e a inserção social (15-17). No campo da saúde bucal, ainda há poucos estudos que abordam essa complexidade e que consideram, de forma integrada, fatores bucais específicos (como perdas dentárias, dor, dificuldades mastigatórias), estilo de vida, morbidades sistêmicas e determinantes sociais na construção da percepção subjetiva da saúde. Dessa forma, identificar os fatores associados a uma autoavaliação negativa da saúde bucal entre idosos, considerando múltiplas dimensões, pode significar uma estratégia relevante para fomentar comportamentos saudáveis e subsidiar o planejamento de ações de saúde pública voltadas ao envelhecimento. 

Este estudo tem como objetivo investigar a associação entre fatores sociodemográficos, hábitos de vida, condições de saúde geral e aspectos bucais com a percepção negativa da saúde bucal entre idosos brasileiros. Em razão de sua abrangência populacional, os resultados obtidos podem oferecer subsídios relevantes para a formulação de políticas públicas voltadas à saúde bucal de idosos no Brasil, bem como em outros países que apresentem contextos socioeconômicos semelhantes.

## Métodos 

### Delineamento do estudo

Este estudo apresenta delineamento transversal, de base populacional. Para sua realização, foram utilizados os dados provenientes da mais recente edição da Pesquisa Nacional de Saúde, conduzida no Brasil em 2019.

### Contexto 

A Pesquisa Nacional de Saúde de 2019, realizada pelo Instituto Brasileiro de Geografia e Estatística em parceria com o Ministério da Saúde, teve como finalidade disponibilizar informações atualizadas acerca das condições de saúde da população brasileira. O inquérito contemplou indivíduos com 15 anos ou mais e investigou múltiplos aspectos, incluindo condições de saúde, estilos de vida, acesso e utilização de serviços de saúde, prevalência de doenças, uso de medicamentos e saúde mental. Além disso, forneceu um panorama das condições de vida da população, evidenciando desigualdades regionais e socioeconômicas. A coleta de dados ocorreu entre agosto de 2019 e março de 2020.

### Participantes 

 A Pesquisa Nacional de Saúde foi conduzida por meio de entrevistas com residentes de 15 anos ou mais, em domicílios localizados tanto em áreas urbanas quanto rurais, contemplando todas as macrorregiões do país. Para este estudo, entretanto, considerou-se exclusivamente a população idosa, definida como indivíduos com 60 anos ou mais, que responderam à questão referente à autopercepção da saúde bucal. Os detalhes acerca do cálculo amostral encontram-se descritos na metodologia da Pesquisa Nacional de Saúde (18).

### Variáveis 

Para a identificação dos idosos que avaliaram negativamente sua saúde bucal, era necessário que os indivíduos relatassem, de maneira subjetiva, que a percebiam como ”ruim” ou “muito ruim” a partir do questionamento: “Em geral, como o senhor(a) avalia sua saúde bucal (dentes e gengiva)?”. Aqueles idosos que responderam a opções “muito boa”, “boa” ou “regular” foram categorizados em uma autopercepção de saúde bucal positiva. 

As variáveis sociodemográficas foram representadas por: sexo, idade, raça/cor da pele, estado civil, escolaridade, plano de saúde e plano odontológico. As de estilo de vida analisadas foram: tabagismo e uso de bebida alcoólica. A variável analisada como indicadora da saúde geral dos idosos foi a presença de duas ou mais doenças crônicas em um mesmo indivíduo (multimorbidade). As variáveis relacionadas à saúde bucal compreenderam a frequência de escovação, a ocorrência de edentulismo e a dificuldade de alimentação decorrente de problemas bucais. Todas as variáveis independentes foram obtidas a partir do questionário da Pesquisa Nacional de Saúde.

### Fonte de dados e mensuração

Este estudo utilizou informações secundárias da Pesquisa Nacional de Saúde, realizada no Brasil em 2019. As informações estão disponíveis em: https://www.pns.icict.fiocruz.br/bases-de-dados/ (19). Além disso, o banco de dados utilizado nesta pesquisa está disponível em: https://drive.google.com/file/d/1VDjasU9Wrteyqfme5LmeCIAX45Z0QrXs/view?usp=sharing.

### Controle de viés

 O controle de viés neste estudo foi realizado com base na estratégia de amostragem da Pesquisa Nacional de Saúde, a qual considerou as diferentes probabilidades de seleção e as especificidades do desenho amostral complexo. Para esse fim, foram atribuídos pesos amostrais aos domicílios e aos indivíduos incluídos, sendo o peso final calculado a partir do inverso das probabilidades de seleção em cada etapa do plano amostral, com ajustes adicionais para não resposta e calibração segundo os totais populacionais. Além disso, por se tratar de uma amostra obtida por conglomerados, empregou-se software estatístico apropriado, que incorporou os efeitos da estratificação e da agregação dos dados na estimação dos indicadores e na determinação das medidas de precisão associadas.

### Tamanho do estudo

A Pesquisa Nacional de Saúde foi realizada em todo o território brasileiro, cobrindo todas as suas macrorregiões, incluindo-se áreas urbanas e rurais, além de englobar domicílios nas capitais e nas regiões metropolitanas. Através da sua ampla cobertura e metodologia detalhada, a pesquisa oferece uma visão abrangente sobre as condições de saúde da população brasileira.

### Métodos estatísticos

A análise dos dados foi realizada no software Statistical Package for the Social Sciences (SPSS), versão 22.0. Inicialmente, procedeu-se à distribuição de frequência das variáveis dependentes e independentes para a elaboração das tabelas. Em seguida, foi utilizado o teste do qui-quadrado com o fim de analisar a relação entre a autopercepção negativa da saúde bucal e variáveis de natureza sociodemográfica, relacionadas ao estilo de vida, à condição de saúde geral e aos aspectos de saúde bucal.

 Também foram calculadas as razões de prevalência brutas e seus respectivos intervalos de confiança de 95%. As variáveis com p-valor<0,200 foram selecionadas para a avaliação de multicolinearidade. Considerando-se o tamanho amostral, definiu-se como critério de multicolinearidade um p-valor≤0,001 entre variáveis independentes, sendo que aquelas fortemente associadas entre si não foram incluídas simultaneamente no modelo multivariado. A análise multivariada foi conduzida por meio de regressão múltipla de Poisson, com estimativa das razões de prevalência ajustadas e respectivos intervalos de confiança de 95%. Embora o modelo forneça como resultado a Incidence Rate Ratio, em estudos transversais com desfecho prevalente, essa estimativa tem interpretação equivalente à razão de prevalência. Todas as análises consideraram o efeito do plano amostral, os pesos de pós-estratificação e as taxas de não resposta.

## Resultados 

A amostra do estudo foi composta por 22.728 idosos, com idades entre 60 e 112 anos, e média etária de 70,1 anos (±7,0). Verificou-se que a maioria dos participantes era do sexo feminino (56,7%), tinha entre 60 e 69 anos (55,4%), se autodeclarava branca (51,3%), era casada (51,1%), alfabetizada (80,7%), não possuía plano de saúde (70,7%), não era fumante (88,1%) e não fazia uso de bebidas alcoólicas (72,6%). Além disso, 60,4% apresentavam duas ou mais doenças crônicas (multimorbidade), 99,6% relataram escovação dental diária, 91,9% não tinham plano odontológico, 96,5% não referiam dificuldade de alimentação devido a problemas bucais e 68,0% não eram totalmente edêntulos.

A prevalência de autopercepção negativa da saúde bucal foi de 33,2%. A distribuição das variáveis independentes e suas associações com a autopercepção da saúde bucal na análise univariada estão apresentadas na Tabela 1. Considerando essa análise preliminar e a verificação de multicolinearidade entre as variáveis, “idade”, “estado civil”, “plano de saúde” e “uso de bebida alcoólica” foram excluídos do modelo de regressão múltipla de Poisson, em razão da elevada correlação com outros fatores. Na análise multivariada, constatou-se associação significativa da autopercepção negativa da saúde bucal com indivíduos do sexo masculino, pessoas autodeclaradas negras, idosos não alfabetizados, aqueles com multimorbidade, os que não realizavam escovação dental diária, indivíduos sem plano odontológico, idosos com dificuldade de alimentação e aqueles que não eram totalmente edêntulos (Tabela 2).

**Tabela 2:** Razões de prevalência (RP) ajustadas e intervalos de confiança de 95% (IC95%) da autopercepção negativa de saúde bucal em idosos pelas variáveis do estudo (análise multivariada). Brasil, 2019 (n=22.728)

Variável	RP (IC95%)	p-valor
Sexo	1,03	<0,001
Masculino	(1,02; 1,04)	
Feminino	1,00	
**Raça/cor de pele^a^**		<0,001
Branco	1,00	
Pardo	1,03 (1,02; 1,03)	
Preto	1,04 (1,03; 1,05)	
Outros	1,01 (1,00; 1,02)	
Escolaridade	1,03	<0,001
Não alfabetizado	(1,02; 1,05)	
Alfabetizado	1,00	
Tabagismo	1,00	0,736
Sim	(0,99; 1,02)	
Não	1,00	
**Presença de multimorbidade^a^**	1,03	<0,001
Sim	(1,02; 1,03)	
Não	1,00	
**Frequência de escovação^a^**	1,14	<0,001
Não escova todos os dias	(1,07; 1,22)	
Pelo menos 1 vez ao dia	1,00	
**Plano odontológico**	1,05	<0,001
Não	(1,04; 1,06)	
Sim	1,00	
**Dificuldade de se alimentar^a^**	1,34	<0,001
Intenso ou muito intenso	(1,31; 1,38)	
Pouca ou nenhuma	1,00	
**Edentulismo total**	1,04	<0,001
Não	(1,03; 1,05)	
Sim		

^a^A diferença para o número total de casos (100,0%) corresponde ao número de casos ignorados, em branco ou com preenchimento ausente no banco de dados da Pesquisa Nacional de Saúde.

**Tabela 1:** Razões de prevalência (RP) brutas e intervalos de confiança de 95% (IC95%) da autopercepção negativa de saúde bucal em idosos pelas variáveis do estudo (análise bivariada). Brasil, 2019 (n=22.728)

	Autopercepção negativa de saúde bucal	Autopercepção positiva de saúde bucal		
Variável	n (%)	n (%)	RP (IC95%)	p-valor
Sexo			1,10	<0,001
Masculino	3.932 (38,6)	7.509 (61,4)	(1,07; 1,13)	
Feminino	4.073 (32,5)	8.462 (67,5)	1,00	
**Faixa etária** (anos)			1,03	0,005
60-69	4.412 (35,1)	8.143 (64,9)	1,00	
70-79	2.469 (34,5)	4.688 (65,5)	(0,93; 0,99)	
≥80	1.124 (37,3)	1.892 (62,7)	(0,99; 1,07)	
**Raça/cor de pele^a^**				<0,001
Branco	3.080 (31,1)	6.821 (68,9)	1,00	
Pardo	3.785 (37,8)	6.216 (62,2)	1,11 (1,08; 1,14)	
Preto	997 (40,6)	1.458 (59,4)	1,16 (1,10; 1,22)	
Outros	142 (38,5)	227 (61,5)	1,10 (0,98; 1,24)	
**Estado civil**				<0,001
Solteiro	1.646 (39,1)	2.564 (60,9)	1,00	
Casado	3.432 (34,5)	6.514 (65,5)	0,91 (0,87; 0,95)	
Divorciado	859 (34,5)	1.629 (65,5)	0,91 (0,86; 0,96)	
Viúvo	2.068 (34,0)	4.016 (66,0)	0,90 (0,86; 0,94)	
Escolaridade			1,29	<0,001
Não alfabetizado	2.314 (43,0)	3.069 (57,0)	(1,21; 1,37)	
Alfabetizado	5.691 (32,8)	11.654 (67,2)	1,00	
**Plano de saúde**			1,46	<0,001
Não	6.526 (38,6)	10.365 (61,4)	(1,35; 1,57)	
Sim	1.479 (25,3)	4.358 (74,7)	1,00	
Tabagismo			1,09	0,050
Sim	1.025 (38,2)	1.655 (61,8)	(1,00; 1,91)	
Não	6.980 (34,8)	13.068 (65,2)	1,00	
**Uso de bebida alcoólica**			1,05	0,017
Sim	1.835 (32,6)	3.797 (67,4)	(1,01; 1,09)	
Não	6.170 (36,1)	10.926 (63,9)	1,00	
**Presença de multimorbidade^a^**			1,20	<0,001
Sim	4.665 (37,0)	7.953 (63,0)	(1,13; 1,27)	
Não	2.913 (32,0)	6.194 (68,0)	1,00	
**Frequência de escovação^a^**			1,89	<0,001
Não escova todos os dias	78 (61,4)	49 (38,6)	(1,54; 2,31)	
Pelo menos 1 vez ao dia	7.343 (34,1)	14.171 (65,9)	1,00	
**Plano odontológico**			1,34	<0,001
Não	7.534 (36,0)	13.394 (64,0)	(1,19; 1,52)	
Sim	471 (26,2)	1.329 (73,8)	1,00	
**Dificuldade de se alimentar^a^**			2,63	<0,001
Intenso ou muito intenso	660 (75,8)	211 (24,2)	(2,45; 2,82)	
Pouca ou nenhuma	6.154 (30,5)	14.023 (69,5)	1,00	
**Edentulismo total**			1,09	<0,001
Não	5.654 (36,4)	9.879 (63,6)	(1,06; 1,12)	
Sim	2.351 (32,7)	4.844 (67,3)	1,00	

^a^A diferença para o número total de casos (100,0%) corresponde ao número de casos ignorados, em branco ou com preenchimento ausente no banco de dados da Pesquisa Nacional de Saúde.

## Discussão 

A percepção da saúde bucal como “ruim” ou “muito ruim” esteve associada a diversas características individuais entre idosos brasileiros. Essa autopercepção negativa foi mais frequente entre homens, pessoas pretas, indivíduos não alfabetizados, com duas ou mais doenças crônicas, sem plano odontológico, que não escovam os dentes todos os dias, que ainda possuem dentes na boca e entre aqueles que relataram dificuldade para se alimentar. Esses achados evidenciam a influência de fatores sociodemográficos, condições sistêmicas, comportamentos de higiene bucal e limitações funcionais na construção da percepção subjetiva da saúde bucal nessa população.

Entre as principais forças deste estudo, destaca-se a utilização de dados de base populacional e com representatividade nacional, o que confere validade externa aos resultados. A adoção de métodos analíticos robustos e o ajuste para o desenho amostral complexo aumentam a confiabilidade dos achados. Como limitação, ressalta-se o delineamento transversal, que não permite estabelecer relações de causalidade. Mesmo assim, os dados fornecem subsídios relevantes que podem subsidiar a implementação de estratégias preventivas voltadas à redução das condições adversas de saúde bucal em idosos no Brasil, bem como em outros países que apresentem perfil socioeconômico e cultural semelhante.

 A prevalência de idosos que avaliaram negativamente sua saúde bucal foi de 33,2%. Esse resultado é motivo de preocupação, pois alterações bucais nessa fase da vida não apenas comprometem funções essenciais, como a mastigação e a nutrição, mas também afetam aspectos emocionais, sociais e estéticos, interferindo de maneira significativa na qualidade de vida (20-24). Comparando-se com os dados do SBBrasil 2010, no qual 28,2% dos idosos apresentaram autopercepção negativa, observa-se um crescimento nesse indicador. Esse cenário reforça a importância de intensificar programas de atenção preventiva e ampliar a oferta de serviços especializados voltados ao cuidado da população idosa.

A análise ajustada evidenciou que homens tendem a relatar percepção mais negativa em relação à saúde bucal do que as mulheres. Isso pode estar associado ao fato de que o público feminino, de modo geral, adota comportamentos de maior cuidado com a saúde, realiza consultas médicas e odontológicas com mais frequência e valoriza práticas de autocuidado. A menor adesão dos homens a esses serviços pode explicar sua maior vulnerabilidade a doenças como cárie e periodontite (25). Além disso, verificou-se que a percepção negativa foi mais frequente entre idosos de cor/raça parda e, sobretudo, preta. Esse achado encontra respaldo em investigações internacionais que apontam forte associação entre desigualdades raciais e piores condições de saúde bucal (26). Em particular, idosos pretos têm maior probabilidade de perda dentária quando comparados a idosos brancos (26), realidade que pode ser agravada por condições socioeconômicas menos favoráveis e menor acesso a cuidados odontológicos.

Também foi constatada associação entre o analfabetismo e a percepção desfavorável da saúde bucal. A ausência de habilidades de leitura e escrita pode restringir o entendimento de informações preventivas e dificultar a adoção de práticas de autocuidado, aumentando a chance de desenvolvimento de cáries e doenças periodontais (27-30). Pesquisas transversais no Brasil já indicaram que pessoas com menor escolaridade utilizam com menor frequência os serviços odontológicos (29,31). Ademais, idosos com baixa escolaridade apresentam mais frequentemente acúmulo de biofilme e alterações de mucosa bucal, reflexos de práticas deficientes de higiene oral (25). Outro aspecto a ser destacado é o baixo letramento em saúde bucal, que se traduz na dificuldade de compreender e aplicar orientações sobre cuidados orais, o que tem sido associado à insatisfação com a saúde bucal (24). Esses fatores indicam a necessidade de desenvolver estratégias educativas adaptadas às condições cognitivas e comunicativas da população idosa.

Por fim, observou-se que a coexistência de duas ou mais doenças crônicas esteve relacionada a uma percepção mais negativa da saúde bucal. A multimorbidade costuma estar associada à maior fragilidade, limitação funcional e maior utilização dos serviços de saúde (32). Nesse contexto, a sobrecarga gerada pelas múltiplas condições clínicas, somada a restrições físicas e psicológicas, pode contribuir para ampliar a percepção negativa da saúde em geral, estendendo esse julgamento também à saúde bucal (30,32).

Variáveis diretamente relacionadas à saúde bucal também estiveram associadas à autopercepção negativa. A ausência de plano odontológico, a falta de escovação diária e a presença de dentes em boca se destacam. Além de refletir condições socioeconômicas desfavoráveis, a ausência de cobertura por plano odontológico limita o acesso a serviços de saúde, podendo resultar em piora das condições bucais e, consequentemente, em autopercepção negativa da saúde bucal. O hábito de não realizar escovação dental diária evidencia práticas de higiene oral inadequadas, aumentando a probabilidade de desenvolvimento de doenças dependentes de biofilme, como cárie e periodontite, o que impacta diretamente na percepção negativa relatada. Ademais, a presença de dentes remanescentes também se associou a uma avaliação desfavorável da saúde bucal, possivelmente devido à ocorrência de dor, uma vez que é comum, entre idosos com dentes naturais, a presença de cáries e enfermidades periodontais (10).

A dificuldade de alimentação decorrente de problemas bucais apresentou forte associação com a autopercepção negativa sobre a saúde bucal. Essa limitação pode resultar de dores dentárias, musculares ou articulares, assim como de dentes em mau estado ou de próteses mal adaptadas, comprometendo a função mastigatória e o conforto ao se alimentar.

Esse conjunto de achados evidencia a inter-relação entre fatores sociais, comportamentais, sistêmicos e bucais na construção da percepção subjetiva da saúde bucal entre idosos. Estudos nacionais e internacionais demonstram que essa percepção é sensível às desigualdades sociais, aos dificultadores de acesso aos serviços de saúde e ao nível de letramento em saúde bucal (24,30). Em diferentes contextos culturais, como observado em estudos conduzidos no Brasil e em outros países da latinos, fatores socioeconômicos e funcionais são determinantes consistentes da autopercepção negativa (30,33).

Os resultados deste estudo reforçam a necessidade de fortalecer a atenção odontológica no Sistema Único de Saúde, com ênfase na ampliação do acesso a próteses dentárias, em ações educativas e no atendimento domiciliar. Estratégias intersetoriais que integrem o cuidado em saúde bucal ao envelhecimento saudável podem contribuir para reduzir as desigualdades e melhorar os indicadores subjetivos e objetivos da saúde bucal na população idosa brasileira (23,33).

Como conclusão, os achados deste estudo sugerem que a autopercepção negativa da saúde bucal entre idosos no Brasil pode estar associada a condições socioeconômicas desfavoráveis e à presença de múltiplas doenças crônicas. Além disso, fatores relacionados à saúde bucal também podem contribuir para essa percepção negativa, como práticas de higiene inadequadas, dificuldade para se alimentar decorrente de condições bucais não saudáveis e presença de dentes remanescentes, fatores que, possivelmente, estiveram associados a experiências prévias de dor.
